# Transposable Prophages in Leptospira: An Ancient, Now Diverse, Group Predominant in Causative Agents of Weil’s Disease

**DOI:** 10.3390/ijms222413434

**Published:** 2021-12-14

**Authors:** Eric Olo Ndela, François Enault, Ariane Toussaint

**Affiliations:** 1 Laboratoire Microorganismes: Genome Environment (LMGE), Université Clermont Auvergne, CNRS, F-63000 Clermont-Ferrand, France; eric.olo_ndela@uca.fr; 2 Microbiologie Cellulaire et Moléculaire, Université Libre de Bruxelles, IBMM-DBM, 12 Rue des Professeurs Jeneer et Brachet, B-6041 Gosselies, Belgium; phagemu1@gmail.com

**Keywords:** leptospira, transposable prophages, phylogeny, evolution

## Abstract

The virome associated with the corkscrew shaped bacterium Leptospira, responsible for Weil’s disease, is scarcely known, and genetic tools available for these bacteria remain limited. To reduce these two issues, potential transposable prophages were searched in *Leptospiraceae* genomes. The 236 predicted transposable prophages were particularly abundant in the most pathogenic leptospiral clade, being potentially involved in the acquisition of virulent traits. According to genomic similarities and phylogenies, these prophages are distantly related to known transposable phages and are organized into six groups, one of them encompassing prophages with unusual TA-TA ends. Interestingly, structural and transposition proteins reconstruct different relationships between groups, suggesting ancestral recombinations. Based on the baseplate phylogeny, two large clades emerge, with specific gene-contents and high sequence divergence reflecting their ancient origin. Despite their high divergence, the size and overall genomic organization of all prophages are very conserved, a testimony to the highly constrained nature of their genomes. Finally, similarities between these prophages and the three known non-transposable phages infecting *L. biflexa*, suggest gene transfer between different Caudovirales inside their leptospiral host, and the possibility to use some of the transposable prophages in that model strain.

## 1. Introduction

In 1886, Adolf Weil (Ariane Toussaint’s great-grand father) first described what is now known as leptospirosis, which he reported as an “acute infectious disease with enlargement of the spleen, jaundice, and nephritis” [1]. This global zoonotic disease currently causes more than 1 million severe cases and 60,000 deaths per year [2], and is associated with agriculture or industrial activities [3]. Its causative agent, the corkscrew shaped bacterium Leptospira, was first identified in 1907 [4] and recognized as the cause of Weil’s disease in 1916 [5]. Bacteria causing this disease were affiliated with the order Leptospirales in 1979 [6], which currently includes only one family (Leptospiraceae) and three genera: *Leptospira*, *Leptonema,* and *Turneriella* [7]. Improvement in isolation procedures and the advent of the genomic era resulted in the sequencing of more than 700 genomes of Leptospiraceae [8,9], allowing for a better understanding of the diversity and evolution of this bacterial family and of its variable virulence. It was recently proposed [10] to organize the *Leptospira* genus in four clades, according to their pathogenic status and phylogeny: (i) a clade termed P1 includes virulent pathogenic strains causing disease in humans and mammals (e.g., *L. interrogans* and *L. weilii* species); (ii) a clade P2 contains ‘intermediate’ species that can cause disease in certain circumstances; and (iii) two clades S1 and S2 made of saprophytes, i.e., free-living environmental microorganisms not known to cause disease, mostly isolated from soil or surface waters. The P1 clade can be further subdivided into a virulent group and low virulence species that may represent an ancestral lineage of pathogens and a secondary passive reservoir of Leptospira present in soils (named P1-virulent and P1-low-virulent [11]). Species in the P1-virulent group have a higher and scattered GC content, a more open pangenome, with many genes specifically found in single species, a lower coding ratio, and a higher number of pseudogenes and signature genes of horizontal gene transfer such as IS transposases [11].

Molecular techniques for the genetic analysis of Leptospira remain very limited [12]. Mutants, a few plasmids, and only three phages are available for a few model strains. Putative prophages have been detected in several leptospiral sequenced genomes [9,13], among which there are potential transposable prophages. *Escherichia coli* phage Mu and *Pseudomonas aeruginosa* phage D3112 illustrate the potential offered by transposable phages for developing powerful genetic tools. Mini-Mu’s and mini-D3112’s have been engineered to remove the lethal phage functions but leave intact the capacity to transpose. They have proved very useful for, e.g., insertional mutagenesis and chromosome mobilization by conjugative plasmids (including broad host range IncP plasmids) and the building of genetic maps in a range of bacterial species [14,15,16,17]. In theory, similar tools could be developed for the many other phyla in which such phages and/or prophages exist [18]. The large number of leptospiral genomes now available offers the opportunity to identify candidate full-length transposable prophages, which, in the future, could help create tools adapted to the genetic and molecular studies for the model *L. biflexa* strains and other Leptospira strains.

*Escherichia coli* (*E. coli*) virus Mu and *Pseudomonas aeruginosa* (*P. aeruginosa*) virus B3 are the paradigm transposable phages, which are still described as multiple independent genera among the Myoviridae (Mu) and Siphoviridae (B3). However, because of their conserved and characteristic genome length, gene content, and replication and packaging mechanisms, it has been proposed to group them into a single family, the Saltoviridae, in the order Caudovirales [19]. Their genome is replicated via successive rounds of replicative transposition (illustration available at https://viralzone.expasy.org/4017, accessed on 1 April 2021), a mechanism that produces insertion mutations, inversions, and deletions at more or less random sites in the host genome, and replicon fusions between resident plasmids and the chromosome. This leads to a profound reorganization of the host genome and far more diverse horizontal gene exchanges than promoted by any other type of phage [18]. Packaging of viral-genome copies into viral particles proceeds directly from the randomly inserted replicas, generating viral DNAs with short segments of the flanking host DNA, the variable ends (illustration available at https://viralzone.expasy.org/4277, accessed on 1 April 2021, reviewed in [16]). The biochemistry of the sequential molecular steps of Mu replicative transposition has been deciphered in vitro, explaining how Mu integration generates a five-base-pair duplication at the insertion target site. The transposase cuts the two target DNA strands five bases apart, and the resulting gap is filled during the replication of the Mu insert from the 3′-OH overhangs [20]. Phage B3 generates a 6 bp target site duplication, which is supposed to result from its transposase cutting six bases instead of five bases apart. The 5/6 bp duplication provides a recognizable scar of the integration event, which, with a relatively well conserved genome length of 36–39 Kb and the presence of the transposition functions, are key features that have been used to identify transposable prophages in their host genomic sequences in a wide range of bacterial species [19,21,22,23]. Here, we identify 236 potential transposable prophages in genomes of Leptospiraceae, and group them at different scales using genome information and various protein phylogenies, in order to have a better idea of their diversity and try to reconstruct their evolutionary history.

## 2. Results

### 2.1. Searching for Transposable Prophages in Leptospira weilii

**Figure 1:**
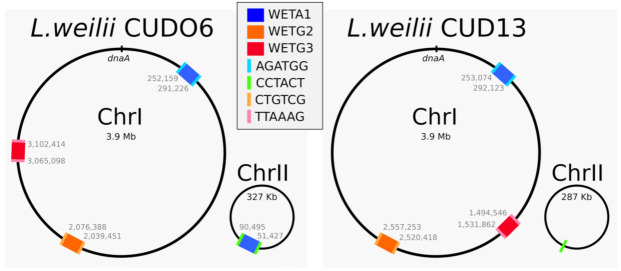
Location of the seven predicted prophages in *L. weilii* CUDO6 and CUD13. The different copies of the three distinct prophages WETA1, WETG2, and WETG3 are colored, as well as the sequences of the flanking 6 bp repeats. See text for details.

Existing prophage prediction algorithms do not reliably predict transposable prophages, often missing one or both ends. This is most likely due to the presence of a rather long, poorly conserved region, the SEE semi-essential region, coding for small proteins with uncharacterized functions. Nevertheless, many such prophages have been manually predicted in different types of bacteria, using similarity searches with four conserved proteins encoded by both phage Mu and B3: the transposase TnpA, the transposition target binding ATPase TnpB, the late regulator Mor/C, and the GemA protein of unknown function [19,22,23]. The Tn*552* transposase, which is related to B3 TnpA, was added here to this set. These nine proteins were first compared to proteins from *Leptospira weilii* genomes available in RefSeq. No proteins from *L. weilii* were similar to Mu proteins, but two closely related *L. weilii* strains, CUDO6 and CUD13, had hits to B3 and Tn*552* transposases and to B3 TnpB. These putative transposases were found at the same location in the chromosome sequences (Chr I) of CUDO6 and CUD13 and in the large plasmid (Chr II) of CUDO6 but not of CUD13. The Chr II sequences of CUDO6 and CUD13 were found to be almost identical except from a 39,069 bp insertion in CUDO6 (positions 51,427 to 90,495), the identified transposase proteins being in this inserted sequence. Furthermore, inspection of the gene annotations in this 39,069 bp region revealed the presence of several phage related proteins, pointing to a putative prophage (in blue in Figure 1). Position 90,496 in CUDO6 Chr II corresponds to position 51,383 in CUD13 Chr II, where it is adjacent to the 6 bp sequence CCTACT. In CUDO6 Chr II, these six base pairs are directly repeated at each end of the 39,069 bp insertion, a footprint for a transposition event generated by a B3/Tn*552* family transposase (in green in Figure 1). However, contrary to the usual TG-CA inverted repeat conserved at the ends of all Mu and B3-like phage and prophage DNA described so far [18], this new putative prophage had a TA direct repeat at its ends. The two regions where a transposase was identified in CUDO6 and CUD13 chromosomes were identical to this 39,069 bp predicted prophage in CUDO6 plasmid, which could have originated from the transposition of a chromosomal copy. In these two Chr I regions, this putative prophage (in blue in Figure 1) was also flanked by a 6 bp direct repeat (AGATGG in both strains), only one copy of this 6 bp sequence being found in other *L. weilii* genomes in which this prophage is absent. This first predicted prophage is referred to as WETA1 (for weilii TA-TA prophage number 1) in all analysis below.

### 2.2. Predicting Transposable Prophages in Leptospiraceae

To expand the search of possibly functional transposable prophages, the predicted WETA1 transposase protein sequence was used to search Leptospiraceae genomes available in the RefSeq repository. As many as 345 putative transposases were uncovered from 330 Leptospiraceae chromosomes and plasmids (amino acid identity percent ranging from 23 to 97%). Contigs shorter than 35 Kb were discarded, as well as contigs containing very long stretches of phage genes, possibly the result of assembly problems and for which defining precise prophage ends would be almost impossible (Table S1). The remaining 236 contigs (Table S2) were good candidates as they were longer than 35 Kb, contained a gene similar to the transposase of WETA1 and other genes similar to transposable phages. Complete prophages and their exact ends were searched in these 236 contigs. However, the 6 bp repeat scar of a transposition event could not be directly identified as repeated 6 bp long sequences are numerous in genomes. Thus, contigs that coded for identical transposase proteins were aligned at the nucleotide level, considering that they should share identical or very similar prophage ends since these contain the transposase binding and cleavage sites. Distinct flanking regions of these prophage pairs allowed to identify both prophage ends, which were then inspected manually to find a flanking 6 bp direct repeat. This strategy identified 97 full length prophages with their 6 bp repeat transposition scar. Proteins encoded by these complete prophages were then used to refine the ends of the 139 remaining prophages.

### 2.3. The Predicted Prophages Predominate in Leptospira Pathogenic Strains

The Leptospiraceae family contains three genera, *Leptospira*, *Leptonema,* and *Turneriella*. The 236 putative prophages defined above occur in thirteen different species of the *Leptospira* genus and only once in another genus, *Leptonema illini* (two bacterial genomes, each with one copy of the same prophage). This over-abundance of prophages in *Leptospira* could be explained by the fact that only two and one bacterial genomes are currently available in RefSeq for the *Leptonema* and *Turneriella* genera, respectively. The thirteen leptospiral species with at least one transposable prophage were distributed across the leptospiral phylogeny and belonged to four of the five leptospiral clades [10,11]: 215 prophages were found in the P1-virulent pathogens clade, three in the P1-low-virulent pathogens clade, five in the ‘intermediates’ clade P2, four in the ‘saprophytes’ clade S1, and none in the clade S2. However, the fact that human pathogens are more studied and sequenced than environmental Leptospira could explain these results. We thus estimated the number of bacterial genomes sequenced for each species, and indeed, only five leptospiral genomes of the ‘saprophytes’ clade S2 were currently available, a trivial reason explaining the absence of detected prophages in this clade (Table S3). Yet, at least 31 genomes were available in each of the other four clades, and the proportion of genomes with at least one prophage was different inside these four clades. Indeed, the proportion of genomes with one or more prophages was 6%, 10%, 13%, and 26% for the S1, P1-low-virulent, P2, and P1-virulent clades, respectively. In addition, the number of prophages in individual strains was also higher in P1-virulent strains, some of which harbor more than one prophage (average of 1.5 prophage per strain). *Leptospira weilii* was the most colonized species, 63% of the strains containing at least one prophage, with an average of almost two prophages per strain, and sometimes up to four prophages as in CUDO6 (Figure 1).

### 2.4. Sequence Comparison between Predicted Prophages

#### 2.4.1. Identifying (Almost) Identical Prophages

The 236 predicted prophage DNA sequences were compared and 80 were found to be almost identical to at least one other sequence (>99% nucleotide identity). Identical prophages always resided in bacterial hosts of the same species. Within groups of identical prophages, most were found in closely related strains isolated from similar geographical locations, but in fifteen cases they resided in bacteria isolated from different orders of mammals (Homo sapiens and Chiroptera, Rodentia, Artiodactyla or Afrosoricida). As leptospirosis is a zoonosis, finding closely related strains (and thus closely related prophages) in humans and in different mammal species is not surprising (Table S2). In addition, the same prophage is sometimes inserted at an identical location flanked by the same 6 bp repeat in different strains, as it is the case in *L. interrogans* strains 56,662 and 56,652 that both contained an identical TA-TA prophage; although, these strains were isolated from rodent (reed vole) and human samples, respectively, and in two different Chinese provinces more than ten years apart. Finally, six individual strains contained several copies of the same prophage (Table S2). These copies are all in separate contigs, as for WETA1 in CUD06 Chr I and II. These numbers are certainly not a correct representation of the occurrence of duplicated copies of the same prophage in a given strain, since as mentioned above, genome assembly tends to put several copies in short contigs missing the ends, which we set aside for this analysis. The 80 prophages identified as duplicates were removed to compile a final set of 156 distinct prophages used in further analysis. As many as 74 of these 156 prophages can be considered as full-length, potentially active prophages, because their ends, as well as the 6 bp scar of transposition, could be identified (Table S2).

Among these 74 complete prophages, 28 had a TA dinucleotide at each end flanked by a 6 bp direct repeat as WETA1 in *L. weilii* CUDO6 and CUD13, with length between 37,910 and 40,416 bp. The remaining 46 prophages had a slightly shorter size (between 35,432 and 38,824 bp) and the classical TG-CA ends (Table S2). The TA-TA and TG-CA prophages were both identified in four species (*L. interrogans*, *L. santarosai*, *L. weilii,* and *L. kirschneri*) and only TG-CA prophages were detected in *L. oguchi*. As many as seventeen leptospiral strains contained prophages of both TA-TA and TG-CA type. Their whole chromosome being assembled as a unique contig, CUDO6 and CUD13 genomes allow a more precise analysis. Both strains contain two different TG-CA prophages (WETG2 and WETG3) and one TA-TA prophage (WETA1), this last one being in two copies in CUDO6 (see above and Figure 1). It has to be noted that the chromosomal regions that contain the WETG2 and WETG3 prophages are inverted in CUDO6 versus CUD13. The inversion does not seem to have been generated by any of the prophages because identical prophages are each flanked by the same 6 bp repeat and the same host genes in both strains. Hence, the inversion rather results from another type of recombination event.

The structure of the WETA1 transposition protein TnpA was predicted using AlphaFold Predictions [24]. The MuA transposase comes out as the closest relative, including the two N-terminal end-binding domains, the catalytic DDE domain and a small beta-barrel [25], and a helix with a positive stripe near the C-terminal end (Figure S1). The enhancer binding domain, which so far appears specific to Mu, is missing. In addition, structure comparison defines the WETA1 TnpA catalytic domain as D163 D269 E305. A similar analysis was performed on TnpA from one TG-CA prophage, INNN139, using RaptorX [26,27]. MuA transposase also comes out as the closest relative, with the same conserved domains as in the WETA1 protein, missing the enhancer binding portion, the DDE domain being here predicted as D161, D280, and E314, consistent with the most conserved D, D, and E residues among all prophage and phage transposases.

#### 2.4.2. Defining Genera and Species Using Genome Comparison

The 156 prophage nucleotide sequences were compared with VIRIDIC [28], a tool that allows delineation of genera and species using thresholds of respectively 70% and 95% identity, as advocated by the ICTV. Using these standard cutoffs, transposable prophages from Leptospiraceae were grouped into 126 species within 25 genera (Figure S2, Table S2). The 126 viral species only include prophages from the same host species, whereas 10 of the 25 viral genera include prophages from several host species. Phages appear to circulate among *L. weilii*, *L. santarosai*, *L. alexanderi*, *L. borgpetersenii*, *L. mayottensis*, *L. interrogans*, *L. kishneri,* and *L. noguchii*, which are phylogenetically closely related and belong to the P1-virulent group, and *L. stimsonii* belonging to the sub-clade P1-low-virulent. Prophage GC-content is comprised of between 37.7 and 44% except for the *Leptonema* prophage that has a GC-content of 54.7 (54.2 for its host). For 76 of the 126 prophage species, the GC-content could also be obtained for their host contig (Table S2) and the phage GC% was greater and less variable (average of 41.2 and standard deviation of 2.3) than the host’s (avg 38.2, sd 3.7). These differences are mainly due to 26 prophages having a GC-content different from their host (>±5%), found in leptospiral genomes with a low GC-content (~35%). Prophage genera found in different host species had homogeneous GC-content, sometimes different from their host’s. For example, SANN49, SATG48, and NOTG66, three prophages belonging to the same genus, had a similar GC content (42.9, 42.8, and 42 respectively), which was similar to their *L. santarosai* hosts for the two first (41.7 and 42.5), but different from the third host, namely, *L. oguchi* (35.2%).

Considering more distant intergenomic similarities computed by VIRIDIC, prophages are organized into six large groups, even though BYNN151 from *L. bouyouniensis* (a soil isolate), KANN77 from *L. kanakyensis* (also sampled from soil), and UNNN26 (undetermined species isolated from water) were only distantly related to others (Figure S2). One of these six groups is only composed of ILNN158 from *Leptonema illini* and three groups contain TG-CA prophages while all the TA-TA prophages belong to a single group. The last group contains only prophages with no defined ends. As no nucleotide sequence similarity exists between groups, sequence information at the amino acid level was used to further investigate relationships between these six groups.

#### 2.4.3. Defining Sub-Families Using the Species Phylogeny

**Figure 2:**
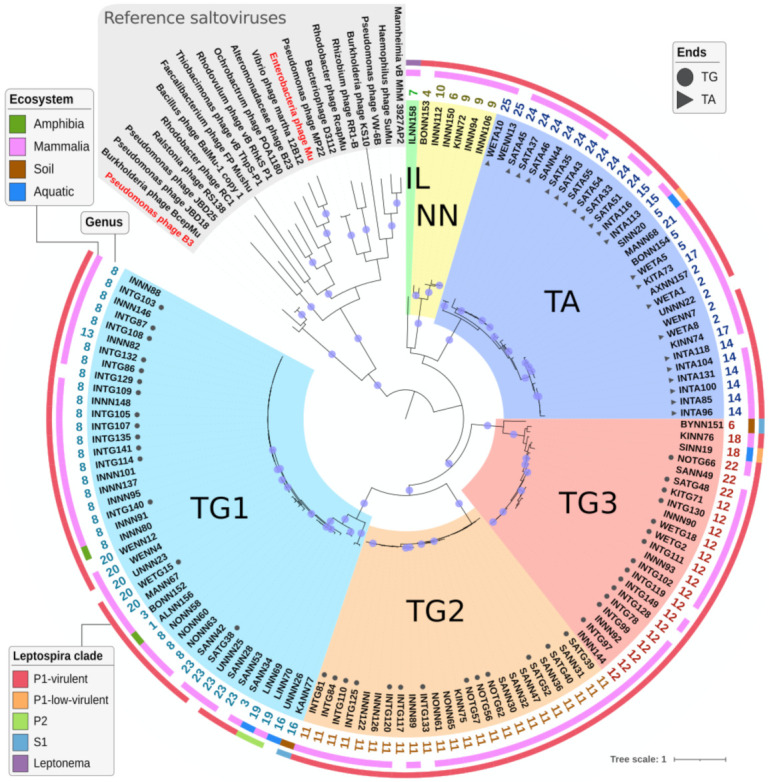
Phylogeny from the concatenation of TnpA and TnpB computed with RAXml. Bootstrap supports greater than 80 are indicated on internal branches by blue circles. Reference transposable phages are colored in grey at the top, with phage Mu and B3 printed in red. The 126 prophages (representative of the 126 prophage species) are separated into six clades, each with a different color, e.g., the TA clade in dark blue. Prophages for which exact ends were determined are indicated with a filled circle or triangle for TG-CA and TA-TA ends, respectively. Displayed as three outer circles are: (i) the genera number of each prophage calculated by VIRIDIC (from 1 to 25), (ii) the ecosystem in which the Leptospiraceae host was isolated, and (iii) the leptospiral clade of the bacteria.

The TnpA transposase and its associated TnpB ATPase are signature proteins of transposable phages. These two proteins were fetched from one representative prophage from each of the 126 species defined by VIRIDIC and from known transposable phages. Considering the phylogeny based on the concatenation of TnpA and TnpB multiple alignments, the 126 prophages form a monophyletic group separated from known transposable phages (Figure 2). As observed for the genomic analysis, the *Leptonema* prophage remains lonely (named IL group), and *Leptospira* prophages form five groups: one with all TA-TA prophages, three with TG-CA prophages, and one with only prophages with undefined ends (respectively named TA, TG1, TG2, TG3, and NN groups). These well-supported groups (bootstraps > 80) are congruent with those defined above with VIRIDIC at the nucleotide level. A group of four transposable phages including B3 is closer to these prophages than the rest of the reference phages. This is not surprising since B3 TnpA was the only core protein from B3 and Mu to show significant similarity when compared to leptospiral genomes. Even though most prophages were extracted from Leptospira isolated from mammals, two, associated to amphibians, are mixed with mammal ones in group TG1. Similarly, two prophages found in strains isolated from water are closely related to mammal ones (SINN20 and SINN19 in TA and TG3 groups). The other four environmental leptospiral prophages, in hosts sampled from water and soil, are clearly separated from animal-associated ones in groups TG1 and TG3. The only two prophages found in saprophyte *Leptospira* (clade S1) are also the only two from soil isolates and are divergent from the rest of the prophages. The two prophages found in P2-intermediate strains are closely related and separated from the rest, whereas no clear difference exists between prophages from the P1-virulent and P1-low-virulent strains. Except for the IL group that contains only one member, the five other prophage groups infect several leptospiral species, respectively, eight, nine, four, seven, and three for the TA, TG1, TG2, TG3, and NN groups, respectively (Figure S3). In addition, most leptospiral species are infected by prophages from different groups, *L. interrogans* being infected by members of the five groups of leptospiral prophages (Figure S3).

#### 2.4.4. Analyzing Gene Content of Transposable (Pro)Phage

**Figure 3:**
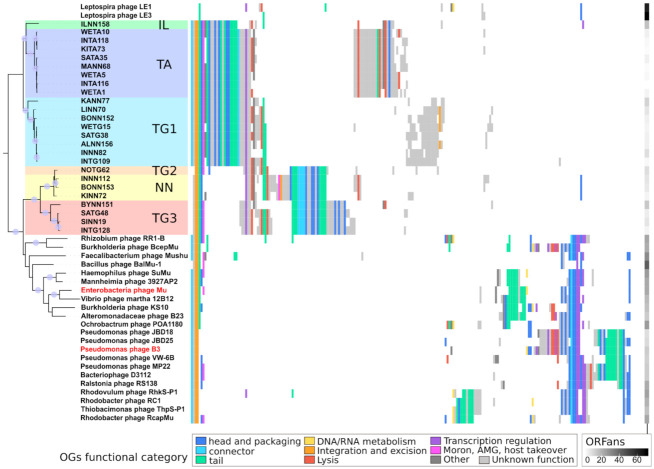
Distribution of orthologous groups (OG) of proteins among reference transposable phages, leptospiral phages and predicted Leptospiraceae prophages. One representative genome was chosen for each (pro)phage genus defined by VIRIDIC. Viruses are organized according to the baseplate subunit protein BW2 phylogeny. Each OG is represented by a column and is colored according to its functional category defined in the PHROG database. The first eight columns of the gene content heatmap represent Ogs conserved in most prophages and known transposable phages and are, from left to right: OG11 (tail completion or neck protein Ne1), OG6 (GemA), OG3 (TnpA), OG2 (TnpB), OG7 (BW2), OG4 (Terminase small subunit), OG5 (Gam), and OG67 (DNA methyltransferase). Ogs containing only one or two proteins were here considered as ORFans and their number in each genome is displayed as a separate column with a grey gradient on the right.

To better understand how Leptospiraceae prophages relate to known transposable phages in terms of gene content, a set of 11,745 proteins was built by combining (i) 8875 proteins predicted from the 156 prophages, (ii) 2625 proteins from 48 reference transposable phages infecting a wide range of bacterial species, and (iii) 245 proteins from the three known *Leptospira biflexa* phages LE1, LE3, and LE4. These 11,745 proteins were clustered into groups of orthologous proteins (OG) using a two-step procedure involving remote homology detection (Table S4). Ogs were then functionally annotated using HMM comparisons to the PHROG database [29]. Considering only the 204 transposable (pro)phages, their 11,500 proteins are organized into 480 Ogs with at least two proteins and 392 singletons. To reduce the redundancy due to the over-representation of some leptospiral strains, a single representative genome was further considered for each of the 47 genera defined using VIRIDIC (25 genera for Leptospiraceae prophages and 22 for reference phages; Tables S2 and S5). TnpA and TnpB transposition/replication proteins are the only ones shared by all genomes of the 47 genera. Alongside these two, GemA, an unknown early function protein, is the only OG present in all but one genera. The other most conserved Ogs (Figure 3; Table S4), shared by most reference and prophage genomes, are: (i) a tail completion also known as neck protein Ne1 (38 genomes out of 47); (ii) a baseplate protein (36 genomes); (iii) a terminase small subunit (33 genomes); and (iv) the Gam protein, which binds linear duplex DNA, conferring protection against RecBCD exonuclease degradation at the onset of viral DNA packaging [30] (30 genomes). Leptospiraceae prophages and known transposable phages have a different set of core genes. Indeed, eight Ogs are found in at least 24 of the 25 Leptospiraceae prophage genera as well as eight Ogs present in at least 21 of the 22 reference genera, with only three Ogs in common (TnpA, TnpB, and GemA). Core proteins for reference phages are composed of the late regulator Mor/C, surprisingly absent from all leptospiral prophages, and of a set of proteins involved in the head and neck formation and DNA packaging (namely, a head maturation protease, portal protein, head–tail adaptor, and neck protein Ne1). Concerning other functional modules such as lysis and tail, their gene contents are more heterogeneous than the prophage ones, possibly reflecting the larger diversity of infected hosts. Accordingly, reference phages infecting closely related hosts have a similar gene content as it is the case for B3 and its relatives infecting pseudomonas (all being Siphoviridae), for Mu and its relatives infecting Gammaproteobacteria (all being Myoviridae), or for phages infecting Rhodobacteraceae (at the bottom of Figure 3). Protein clusters were here built as groups of homologs and not orthologs. Hence, some contain paralogous proteins. Only 3 Ogs conserved in more than 10 phages have a significant number of paralogs (>25% of proteins of the OG): a transcriptional regulator (OG1) that has a paralog in 60 prophage genomes (2 having 3 copies); a likely structural protein (OG17) of unknown function (positioned between sheath and tape measure genes) that has paralogs in TG2 and NN; and an OG of unknown function (OG41) in TG1. All paralogous copies are adjacent on the genomes.

#### 2.4.5. Determining Families Using Phylogeny and Gene Content

A phylogeny was computed for the most conserved structural OG, a baseplate wedge protein (referred to as BW2 in Mu [31]), and was used to organize (pro)phages in a gene content matrix. The six Leptospiraceae prophage groups defined above with the TnpA/TnpB phylogeny are well supported monophyletic groups in the baseplate phylogeny (Figure 3), except that the only genus of the TG2 group is here found among prophages of the NN group. Moreover, the relationships between groups are different in the baseplate and in the transposition proteins phylogeny. Two large sub-trees gather TA, TG1, and IL (in cold colors) and TG2, TG3, and NN (in warm colors) in the baseplate phylogeny, while TG1, TG2, and TG3 were associated using transposition proteins. These two large monophyletic groups, hereafter termed cold and warm groups, are further supported by their gene content. Indeed, seventeen and nineteen Ogs are specific to the cold and warm groups respectively (i.e., present in all but one genome of this group and absent from the other). The seventeen and nineteen specific Ogs represent two different, almost complete, structural modules. Each group of prophages have their own version of major head, portal, head closure, tail completion, tape measure, tail sheath, and baseplate proteins. It has to be noted that OG23 and OG42, two Ogs specific to the cold and warm groups respectively, are annotated as tape measure proteins and exhibit similarity. These two Ogs were not grouped into a single one because the similarity is below the defined threshold (it covers less than 15% of the two proteins). Furthermore, even if no similarities are detected between the remaining 34 specific Ogs, five of them, specific to ‘cold’ prophages, could be indirectly linked to six specific to ‘warm’ prophages, because they were similar to the same PHROG. For example, OG25 and OG43 respectively found only in ‘cold’ and ‘warm’ prophages were both significantly similar to PHROG52, a much more diverse group including 1295 proteins, annotated as ‘tail proteins’. Similarly, eight Ogs containing Mu structural proteins were linked to at least one of the 36 prophage specific Ogs, through similarity to the same PHROG. In addition, even if separated into multiple Ogs, a sheath protein is present in all leptospiral prophages, further indicating that they all harbor a contractile tail, such as the Myoviridae phage Mu.

**Figure 4:**
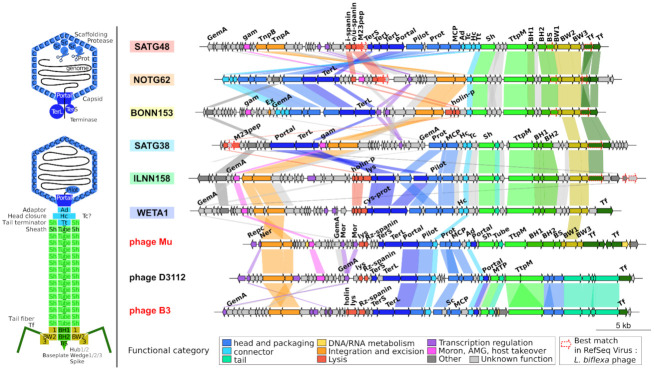
Genomic organization of the predicted prophages. One reference genome was chosen for each of the six prophage clades and reference phage Mu, B3, and D3112 are also shown. Protein coding genes are indicated with arrows showing their transcription orientation and are colored according to their functional category defined in the PHROG database. In addition, proteins whose best match in RefSeqVirus is a *L. biflexa* phage (LE1, LE3, or LE4) are highlighted in red dashed arrows. Similarities between genes from adjacent virus are displayed and were here defined by OG membership. On the left, two schematic representations of the head morphogenesis and final phage Mu structure. TnpA: transposase. TnpB: DNA transposition protein. MCP: major capsid protein; Pilot: Minor head and ejection (pilot) protein; Portal: portal protein; TerL: terminase large subunit; TerS: terminase small subunit; Sc: scaffolding; Prot: head maturation protease; BH, BS, BW: baseplate hub, spike, and wedge proteins; Sh: tail sheath; Tube: tail tube; MTP: major tail protein. TtpM: tail tape measure protein. Tf: tail fiber; SEE: semi-essential region. Ad, Hc, Tc, Tt: head-tail joining. Lys: endolysin; M23pep: metallo-peptidase; holin-p: holin/antiholin pair; and cys-prot: cysteine protease. The location of the tail completion protein (Tc, also known as neck protein Ne1) on the viral particle remains unknown. This figure summarizes Figure S4.

As expected, LE1 and LE3 non-transposable phages have very little similarity with any of the other genomes analyzed (at the top of Figure 3). Only fourteen and five of their respective 81 and 83 proteins were clustered in Ogs with proteins from other phages. They lacked all the above conserved proteins, except a baseplate component. Some proteins from these *L. biflexa* phages have however a closest relative among transposable prophage proteins. When prophage Ogs are compared to NCBI RefSeqVirus proteins (526,375 proteins from 13,778 viruses), a handful are most similar to proteins from these *L. biflexa* Caudovirales: (i) OG16 has its best hit against tail fiber protein from LE3; (ii) OG18 (endolysin) and OG20, adjacent on genomes, are most similar to gp49 and gp51 of LE1; (iii) OG47 is most similar to the baseplate spike of LE1; and (iv) the two genes at the end of BYNN151 and ILNN158, just downstream of the tail fiber protein coding genes, are similar to hypothetical proteins of LE1 (gp46-47) and a hypothetical protein and a metallo-protease (gp80-81), respectively (Figure 4).

### 2.5. Genome Organization of Leptospiraceae Prophages

Known transposable phage genomes are organized into functional modules, expressed sequentially, involved in regulation, replication (by transposition), and building of head and tail structures. These modules are apparent on the genomic maps of Leptospiraceae prophages from the six groups defined earlier and their genomic organization is very stable inside each group (Figure S4). All prophages display a left and a right arm, transcribed in opposite directions, separated by one to three regulatory genes, which most likely regulate the lysis–lysogeny response and the lytic cycle (Figure 4 and Figure S4). This is typical of the B3 genome organization. When present (except in the TG2 group represented here by NOTG62), the *gemA* gene is near one genome end, again, as it is the case in B3. As mentioned earlier, none of the predicted prophage has the paralogous Mor/C genes, the middle and late transcription activators, so far considered as marker genes of transposable phages [21,22]. The replication module (the DDE transposase and the associated ATPase) and the *gam* gene are grouped (except in the NN group) and transcribed opposite from the regulatory genes. This *gam* gene, of the semi-essential SEE region in Mu and B3, is present in almost all the predicted prophages and is often (but not always) located next to a series of short genes of unknown function, which could constitute the SEE region.

The position of head structural genes is variable. The TerS-TerL-Portal block is separated from the other structural genes in the TG1 group as well as the block TerS-TerL-Portal-Pilot-Protease-MCP-adaptors in the NN and TG2 groups. Head and tail modules are always transcribed in the same orientation, with the head genes upstream of the tails in the remaining TA, IL, and TG3 groups. The tail module is at the end of all prophages. Despite the high sequence diversity between prophages of the cold and warm groups, which prevented the clustering of their structural proteins in orthologous groups, various genes could be annotated when compared to PHROG protein families and these identified annotations concern genes that are in the same order in all genomes: the tail sheath (Sh), tube, tail tape measure protein (TtpM), baseplate hub (BH1, BH2), and wedge subunits (BW1, BW2, and BW3) and tail-fiber (Tf) proteins.

Lysis proteins, a key functional module present on all Caudovirales and required for the release of mature viral particles at the end of the lytic cycle, were not directly visible. Three types of proteins together ensure the timely degradation of the host cell wall. The spanins, which appear to bridge the outer and inner membranes, come in two forms. The two component spanins comprise a lipoprotein located in the outer membrane, the o-spanin, and an inner membrane i-spanin with a coiled-coil periplasmic domain. The unitary u-spanins span the periplasm and are anchored in the outer membrane by a C-terminal transmembrane domain and in the outer membrane by a N-terminal lipoprotein domain. The holin/antiholin pair of proteins pierce the inner membrane in due time to allow for the endolysin to access and degrade the peptidoglycan [32]. Using a procedure to specifically identify these lysis functions, a metallo-peptidase with an M23 conserved domain was identified in all TG-CA prophages. This gene might code for an endolysin as in Meiothermus bacteriophage MMP17 where it has been shown to disrupt bacterial cells and exhibit antimicrobial activity against both Gram-negative and Gram-positive pathogenic bacteria [33]. Predictions of holins and spanins appeared in the vicinity of this M23-domain peptidase, supporting the presence of a lysis module at that position. In the TA-TA prophages, a holin/antiholin pair and a predicted cysteine protease, a putative endolysin, reside together in the middle of the genome. Except for those in groups NN and TG1, all prophages have the putative lysis module close to the head module.

Finally, several prophages carry an IS insertion extending their length by 1–2 Kb, e.g., an IS*3* family element in WETA8 and WETA10 (TA group) and SANN28 and WENN9 (TG1 group) and an IS*110* family element in INNN112. Interestingly, apart from its TnpA, INNN139 possesses another protein clustered in the OG3 but located on the left arm of the genome slightly overlapping the major head protein coding gene, likely an IS*481* family element that might disturb the head maturation of this prophage, thus inactivating its ability to produce new virions. The position of the IS near the *terL* gene in WETA8, may also hamper its activity.

## 3. Discussion

### 3.1. A Role in the Virulent Lifestyle Acquisition?

Numerous full length, most likely active, transposable prophages were found in the genomes of bacteria from the Leptospiraceae family. They predominate in the leptospiral P1-virulent clade, both in terms of the proportion of bacterial genomes colonized and of the number of prophages within each colonized strain. This suggests a link between these prophages and the pathogenicity of these bacteria, and is coherent with the abundance of IS transposases discriminating virulent members of the P1-clade from the low-virulent ones [11]. This link is most probably not due to prophage-encoded proteins, similarly to phage-encoded bacterial sigma factors described to modify host phenotype in *Bacillus anthracis* [34]. Here, prophages infecting pathogenic and non-pathogenic Leptospira have very conserved gene content inside each prophage clade, and no gene was found to be specific of virulent Leptospira prophages. Furthermore, the putative lipoprotein Loa22, an OmpA-like protein, known to be necessary for virulence of *L. interrogans* [35] was not found in these prophages. However, transposable prophages are also known to facilitate adaptive evolution of bacterial pathogens within hosts through random integration into the bacterial chromosome and selection of insertional inactivation mutations [36,37]. Thus, a detailed analysis of insertion locations of the prophages identified here could be of great interest, as their recurrent inactivation of a specific gene or transcription factor could impact host phenotype. Finally, these transposable prophages are likely to have an indirect influence on the acquisition of virulence. Indeed, replicating transposable prophages are known to provide a high genome flexibility to their host, which could contribute to the larger number of paralogs in virulent Leptospira [11]. The abundance of transposable prophages in those strains is thus compatible with the hypothesis that by increasing genome plasticity, these mobile elements help bacteria to adapt to a virulent lifestyle.

### 3.2. Complex Relationships: Different Prophages in the Same Host, Different Hosts with the Same Prophages

Many bacterial strains contain two or more, either identical, or different prophages. Several identical prophages in different contigs of a strain, is a strong indication of a transposition activity. Furthermore, prophages detected here are probably far from representing the total population of such sequences in the host species considered, as the presence of several copies in a given bacterial genome will lead to misassembly and to undervalue of the true number of copies. The presence of different transposable prophages in a single host genome is more surprising even if their cohabitation might not be a problem as they do not share any significant nucleotide sequence similarity. However, for different prophages with the same type of ends, complementation between the transposases cannot be excluded. In addition, individual leptospiral strain isolates infecting different eukaryotic hosts sometimes carry identical sets of different prophages inserted at the same chromosomal location, showing that prophages can remain stable for ‘long’ periods of time. Finally, very similar prophages exist (i) in hosts isolated from remote locations, another hint that they remain stable even in terms of sequence variation; and (ii) in different leptospiral species, suggesting that they circulate between bacterial species, and hence could support inter-species horizontal transfer; although, the bacterial species concerned here are closely related. At a larger scale, members of five of the six prophage groups infect several leptospiral species belonging to different leptospiral clades, and most leptospiral species are infected by prophages from different groups (Figure S2). Thus, either these prophages are prone to horizontal transfer between distantly related Leptospira or common ancestors of these prophage groups were already infecting the ancestor of Leptospira.

### 3.3. Prophages with Unusual Genome Ends

The presence of the 6 bp direct repeat, scar of a transposition event, was found in 97 prophages. Even if most prophages have the expected 5′TG-CA3′ genome ends, a subset present in all but one of the leptospiral species analyzed have less canonical TA-TA ends. The case of *L. weilii* CUDO6, which shares with its very close relative CUD13 a TA-TA and two TG-CA prophages (WETA1, WETG2, and WETG3) and gained a copy of WETA1 in its plasmid ChrII is a strong argument for the capacity of this type of prophages to actively transpose. The structure prediction of the TnpA sequence of the the WETA1 prophage showed the end-binding, DDE catalytic and other expected domains present in Mu TnpA, the D, D, and E being the only residues conserved among all TnpA from prophages and phages. There is thus no clear deviation from a standard transposable phage TnpA, and unfortunately, the current understanding of the role of the conserved end base pair is not precise enough to allow for evaluating how the TG to TA and CA to TA transitions might influence recognition and cutting of the prophage ends. Whether this TA-TA type of prophages exist in other bacteria also remains to be investigated.

### 3.4. Transposition and Structural Modules with Similar Yet Different Evolutionary History

The transposition proteins phylogeny allows to delineate six clearly separated prophage groups, and Leptospiraceae prophages form a monophyletic group separated from the known transposable phages, with B3 as the closest relative. Even though these six groups could almost be identified on the baseplate phylogeny, their relationships are different when considering this structural protein. The fact that the evolutionary histories of the transposition and structural genes are not exactly congruent is not unexpected in view of the general combinatorial composition of bacteriophage genomes [38]. It attests of gene transfer events, maybe through recombination. Yet, the incongruencies are mostly apparent from remote branching (i.e., relationships are different between the six groups), probably the consequence of transfer having occurred in a distant past in which these prophages had not diverged too much.

### 3.5. Homologs Are So Divergent That Most Are Not Directly Identifiable

Considering the baseplate phylogeny, prophages are separated into two larger clades: the ‘cold’ clade gathering TA, TG1, and IL prophages and the ‘warm’ clade made of TG2, TG3, and NN prophages, the latter being more closely related to Mu and its relatives than to the ‘cold’ clade. Despite this proximity on the baseplate phylogeny, prophages in the ‘warm’ clade have no structural protein-coding gene in common with Mu-like phages. Prophages have more genes in common between themselves than with any other reference phage, even though most structural genes are specific to either one of the ‘cold’ and ‘warm’ group. Yet, a handful of Ogs specific to the “cold”, “warm”, and Mu-like clades display sequence similarity, although not sufficient to group them. In addition, a significant number of these Ogs were similar to the same PHROGs, which are more diverse orthologous groups as they are made of almost a million proteins from 17,485 (pro)viruses. Considering the position of these genes on the genomes and their length, we thus propose that these sequences are bona fide homologs, having diverged so much that their similarities are no longer detectable, not even when comparing their respective HMM profiles. This result is further evidence of the wide sequence divergence observed in most viral protein families, which remains to be explained. It could arise from lower evolutionary constraints on viral proteins (structural ones, in particular) compared to bacterial proteins, from the ancestral nature of most virus groups, from a higher mutation rate on viral genomes, or from a combination of one or more of these features. This great sequence divergence likely leads to overestimating the number of viral protein families and hence the number of viral groups on Earth.

### 3.6. The Core Genome of Transposable (Pro)Phages Is Limited to … Transposition Genes

Considering these new prophages, the only core proteins of transposable (pro)phages are now down the two transposition proteins TnpA and TnpB. The other three proteins that were present in all known transposable phages, and thus that were considered as essential for such phages, are here found in most but not all new genomes (Gam and GemA) or even absent from all prophages (Mor/C). Whether these genes are indeed absent and dispensable, or are in fact present but with sequence similarities too low to be detected is impossible to conclude. The latter hypothesis is indeed possible as most genes involved in regulation, lysis, and head and tail functional gene modules are clearly identified on genome maps, even though not directly similar to each other as previously mentioned.

### 3.7. The Evolutionary History of Leptospiraceae Transposable Prophages Remains Elusive

The B3-like organization, a left and a right arm separated by regulatory genes, candidates for controlling the lysis–lysogeny switch, is conserved across all prophages despite variation in the relative positions of the integration/replication (TnpA, TnpB), lysis, and head and tail modules in the six groups. Furthermore, transposition proteins of Leptospiraceae prophages are closely related to B3-like phages. Despite these traits in common to the B3-like Siphoviridae, all prophages appear to belong to the Myoviridae family, as they harbor a sheath protein and have (a few) more identifiable homologs with Mu-like Myoviridae. Thus, the origin and evolutionary trajectory of these prophages (and of transposable phages in general) remain difficult to elucidate and additional transposable prophages would help in this regard. In addition, most prophages have a handful of proteins, all involved in either recognition or lysis of the host, whose closest relatives among all Caudovirales are from non-transposable leptospiral phages, indicating that genes directly linked to the host are prone to horizontal transfer between very different types of Caudovirales, further blurring the evolutionary signal.

### 3.8. High Amino Acid Divergence but High Conservation of Genome Length and Organization

Despite a limited number of inversions, the synteny and length (37–39 Kb) of all transposable phages and predicted prophages is remarkably conserved across the wide range of hosts they infect (Figure 4 and Figure S4). These conserved features are even more striking when considering the extreme amino acid divergence noted in transposable phages, as homology is often impossible to identify between prophage groups. Thus, constraints on genomes’ length and organization must be strong and some features previously identified as essential in Mu may be involved. The conservation of the gene order might be a result to the constraints of the sequential expression of genes, described in most well-studied phages like in phage Mu (https://viralzone.expasy.org/4356, accessed on 27 October 2021). The conservation of genome lengths are likely due to constraints of the transposition mechanism, such as the ones potentially imposed by the presence of a gyrase binding site. Indeed, a strong gyrase binding site (SGS) located in the center of the genome [39], which is necessary for efficient Mu replicative transposition [40], allows the integrated Mu DNA to synapse its ends [41] and organize into a stable chromosomal domain [20]. Synapsis of the ends is an essential preparatory step for the assembly of the transpososome, the nucleoprotein complex in which the transposase cleaves Mu ends and ligates them to the new chromosome target site during replication. Such detailed studies do not exist for any other transposable phage. Hence, it is not clear whether gyrase binding sites are a conserved feature of transposable phages. Phages such a s D3112, which code for anti-CRISPR proteins [42] have acquired those genes at the position expected for an SGS (Pato M. personal communication), which may be an indication for the presence of a preferred cleavage site by gyrase, favoring non-homologous recombination at that position. Such a site could be one of the features that constrain the lengths and synteny of these genomes.

## 4. Material and Methods

### 4.1. Prophage Search in the RefSeq Database

Four protein sequences, namely, GemA, Mor, portal, and DDE transposase from phage Mu and B3 were downloaded from the NCBI (respectively NP_050620.1, NP_050621.1, NP_050633.1, NP_050607.1 for Mu and YP_164038.1, YP_164037.1, YP_164068.1, YP_164048.1 for B3) as well as Tn*552* transposase (Accession: X52734.1, CAA36949.1 DDE transposase). These nine proteins were compared to RefSeq proteins encoded by *L. weilii* (BLASTp, bit-score > 50, accessed on 1 January 2021 [43]). This allowed for the identification of the transposase protein of one possibly complete prophage named WETA1. This transposase sequence was then compared to genomes in the Leptospiraceae family (taxid:170) contained in RefSeq Genome (tBLASTn, accessed on 1 March 2021). Genomic contigs carrying a transposase were manually inspected for the presence of additional annotated phage proteins in order to identify potential prophages. First, exact borders of these prophages were tentatively searched when identical transposase protein sequences were found in contigs of different Leptospira strains. Such pairs of contigs were aligned at the nucleotide level and allowed to identify 36–39 Kb segments with conserved 200–400 bp end sequences. Using successive rounds of BLASTn, the 2 bp direct repeat (TA-TA or TG-CA) and the 6 bp flanking direct repeats could then be manually retrieved in these end sequences for 97 putative prophages. The ends of 139 additional putative prophages were then defined using these 97 full-length prophages, by taking 200 bp upstream and downstream of the first and last genes similar to one of these 97 prophages. These 236 predicted prophages were deduplicated using BLASTn (nucleotide identities > 99% over their entire length), leading to a final set of 156 distinct prophages. From these, 74 were part of the 97 complete prophages for which the exact ends were identified.

### 4.2. Genomic Comparisons

These 156 prophage sequences were compared using VIRIDIC [28] to define genera and species. For each prophage, GC percent was computed for its sequence and for its host by taking the rest of the contig out of which it was extracted, only if the remaining sequence was longer than 50 Kb.

### 4.3. Two-Step Protein Clustering

Proteins encoded by these 156 prophages, predicted by Prodigal [44], were combined with those from 48 reference transposable phages and the three available *L. biflexa* phages LE1, LE3, and LE4 retrieved from RefSeq. The resulting set of 11,745 proteins was organized into Orthologous Groups (OGs) in a two-step procedure: (i) all proteins were compared to each other using MMseqs [45] (bit score  ≥50 and reciprocal coverage >50%) and clustered with MCL [46] (inflation 2.0); and (ii) for each protein cluster, a multiple alignment was built using ClustalOmega [47], transformed into an HMM profile and all profiles were compared to each other using HHsearch of the HHsuite toolkit [48] (version 2.0.16, cut-offs: probability ≥90% and coverage ≥50%). Singleton proteins were also individually compared to all these HMM profiles. Using significant similarities, singletons and protein clusters were then again clustered with MCL (inflation 2.0). This second step involving HMM comparisons was then performed a second time to group clusters that were similar, yet not already gathered due to the entangled nature of similarity networks and to the fact that larger and more diverse clusters allow the detection of more distant homologies. Finally, using the same tools, multiple alignments and HMM profiles were computed for all OGs. The OGs HMM profiles were compared to the HMM profiles of the PHROG database [29] (annotation release v2), which contains well annotated protein clusters of prokaryotic viruses. This provided a probable functional annotation for OGs similar with at least one PHROG profile. These putative functions were refined using the Virfam web tool with default homology thresholds [49]. The OGs and their final functional prediction are listed in Table S4.

### 4.4. Phylogenies and Gene Content Matrix

From these OGs, the transposase TnpA, the transposition target binding ATPase TnpB and the baseplate wedge subunit BW2 [31] were identified (OG3, OG2, and OG7, respectively) and their multiple alignments were used to compute phylogenies with RAxML [50] (v8.2.12, 100 bootstrap replicates). Based on the congruency of TnpA and TnpB phylogenies, their respective multiple alignments were concatenated and used to build the phylogeny displayed in Figure 2 (RAxML, 100 bootstrap replicates), and visualized and refined using iTOL [51]. A presence/absence matrix of OGs was generated using the ggplot R package [52] using one representative genome for each genus defined by VIRIDIC (25 genera for the 156 prophages, 22 for known transposable phages and 2 for *L. biflexa* phages, Tables S2 and S5). On this matrix, genera representatives were ordered according to the topology of the baseplate wedge subunit BW2 phylogeny, and visualized and refined with iTOL [51]. In addition, the putative lipoprotein Loa22, an OmpA-like protein, described as a virulence factor in *L. interrogans* [35], was searched using BLASTp (bit-score >50), and no prophage proteins were found similar to Loa22.

### 4.5. Genomic Maps

Genomic maps of one representative genome for each of the six defined prophage clades and three reference transposable phages (Mu, B3, and D3112) were constructed using the genoPlotR package [53]. Plotted genes were colored using their PHROG functional categories and their synteny was identified by OG membership. Annotations were manually refined considering additional information on PHROGs similar to the OGs (e.g., their similarity to PFAM profiles) and on the presence of annotated Mu proteins. For example, the Mu baseplate module was annotated based on its extensive study by Büttner et al. in 2016 [31]. Lysis genes were predicted within the Apollo annotation frame [54]. Briefly, genes were defined taking into account the fact that o-spanins are entirely embedded in i-spanin genes, and translated proteins were compared to dedicated databases, and searched for lipo-boxes and transmembrane domains.

### 4.6. Number of Sequenced Leptospiraceae Species

To estimate the number of genomes available for all Leptospiraceae species, the RpoB protein that encodes the β subunit of bacterial RNA polymerase was used. First, RpoB from *L. interrogans* (serovar Lai 56601: LA_3420) was compared using tBLASTn to all complete Leptospiraceae genomes of the KEGG database to verify that this protein was only found as a single copy in each complete genome. Then, this protein was compared to the genomes in the Leptospiraceae family (taxid:170) contained in RefSeq Genome (tBLASTn, bit-score > 50, accessed on 1 March 2021). The number of significant hits was then computed for all species of this family, and each of the *Leptospira* species was affiliated to one of the five clades (P1-virulent, P1-low-virulent, P2, S1, S2) (Table S3).

### 4.7. Transposase 3D Structure Prediction

A 3D structure prediction was computed for the TnpA sequences of the WETA1 and INNN139 prophages using AlphaFold Wonder Predictions [24] and the RaptorX server [26,27], respectively, which use residue co-variation and deep learning.

## 5. Conclusions

The primary goal of this study was to evaluate the existence of transposable (pro)phages that could be used to derive genetic tools for Leptospiraceae. The set of diverse prophages found here, with characteristics conserved with known transposable phages (e.g., length around 38 Kb, genome organization, randomly integrated viral genome flanked by a short direct repeat of the target site, and DDE transposase and its activator) represent both potential powerful genetic tools and a better understanding of the phage diversity associated with these bacteria. Prophages with new TA-TA ends are especially good candidates to be tested for their ability to transpose in, and eventually infect, *L. biflexa,* and build genetic tools for this model strain such as Mini-B3-like derivatives with functional ends, a selection genetic marker, and expressing the transposase in cis or in trans.

## Data Availability

A GenBank formatted file of the 156 deduplicated prophages is available at https://doi.org/10.5281/zenodo.5592800.

## Supplementary Materials

The following are available online at https://www.mdpi.com/article/10.3390/ijms222413434/s1.
