# Supplementary material for: Transposable Prophages in Leptospira: An Ancient, Now Diverse, Group Predominant in Causative Agents of Weil’s Disease
Source: Int J Mol Sci. 2021 Dec 14;22(24):13434. doi: 10.3390/ijms222413434 (PMC8705779; doi:10.3390/ijms222413434)
Supplement: Supplementary file 1 [file ijms-22-13434-s001.zip › Figure S1¿CS3.pdf]

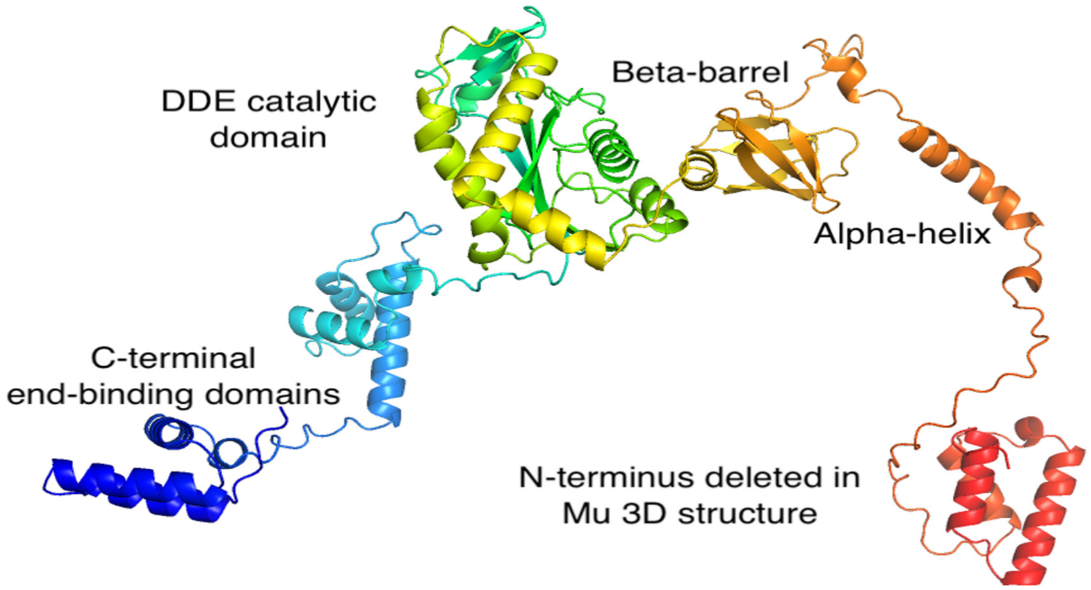

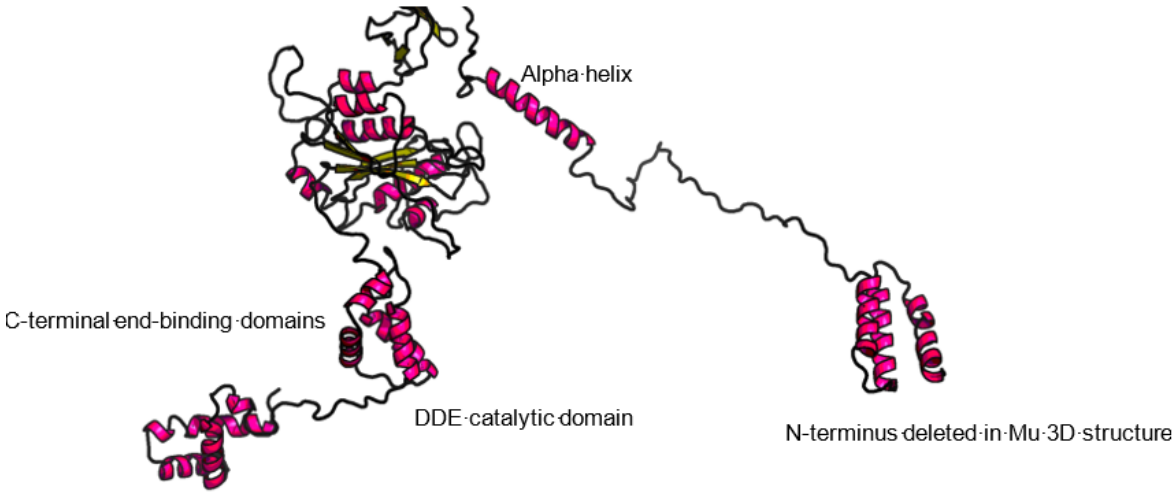

Figure S1. 3D structure prediction of the transposition protein TnpA of prophages WETA1 (TA-TA ends; upper) and INNN139 (TG-CA ends; lower).

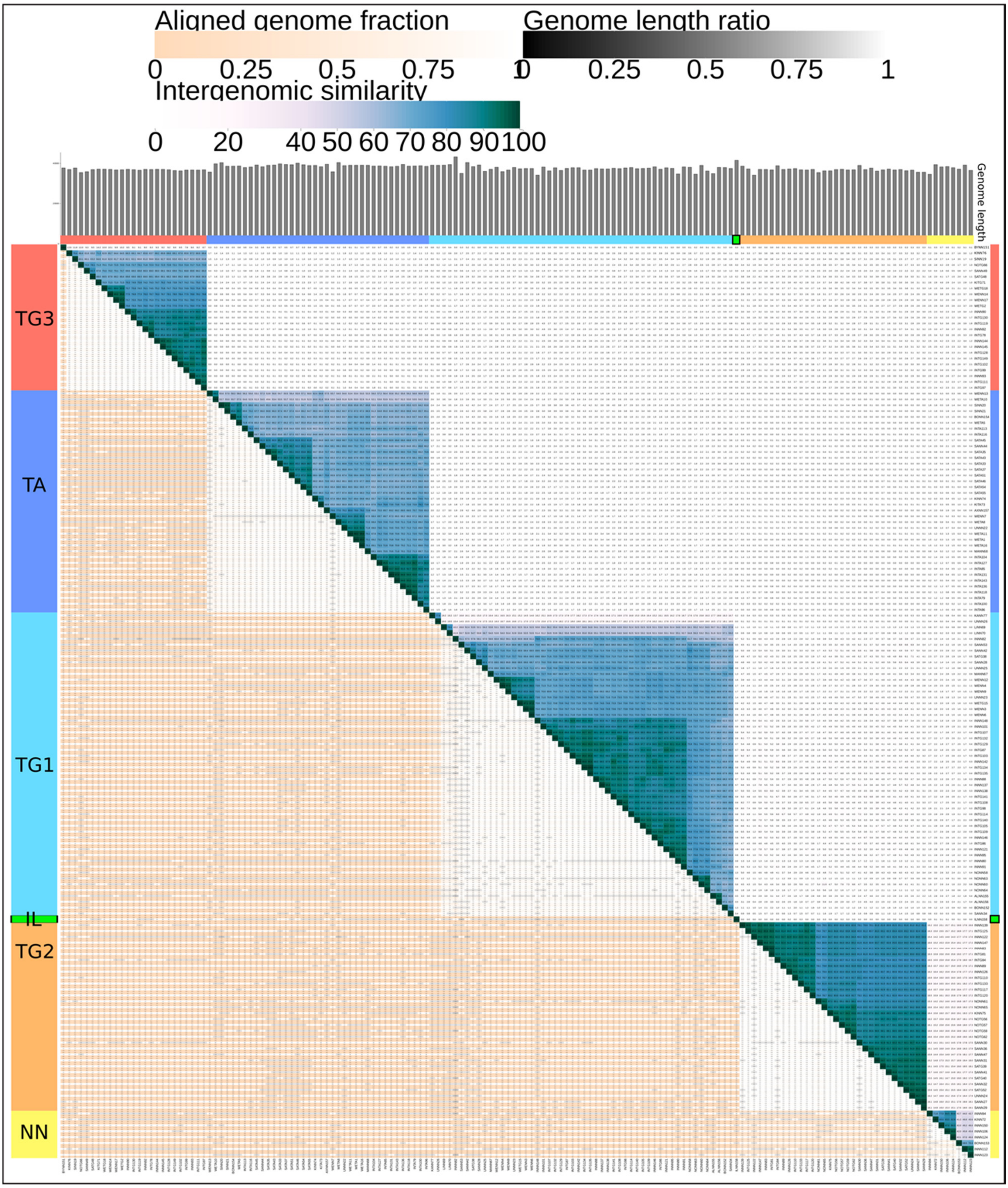

Figure S2. Comparison and clustering of selected predictions by VIRIDIC. The heatmap shows intergenomic similarities (right half) and alignment indicators (left half and top). Right half: numbers represent the similarity values for each pair of genomes, the darker the color the higher the similarity value. Left half: three indicator values are shown for each genome pair. From top to bottom: aligned fraction genome 1 (for the genome found in this row), genome length ratio (for the two genomes in this pair) and aligned fraction genome 2 (for the genome found in this column). The lighter the colors, the bigger the fractions of the genomes were aligned. Overall, genome pairs with high similarity values also have high genome length ratios.

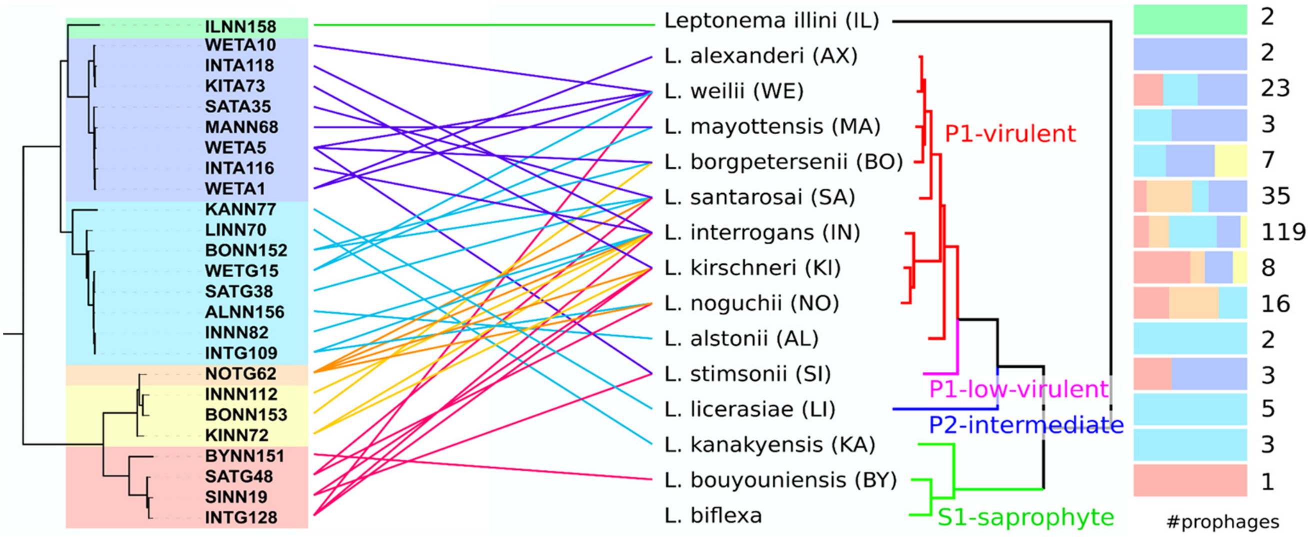

Figure S3. Tanglegram of phylogenetic trees of transposable prophage genera and of *Leptospiraceae* species. One representative phage was selected for each genus and a phylogeny was built based on the baseplate subunit BW2 phylogeny as in Figure 3. The phylogeny of species from the *Leptospiraceae* family was retrieved from by Vincent et al. 2019 [10]. Prophages are linked to *Leptospira* or *Leptonema* species when found in its genomes, the proportion of each of the 6 prophage group is mapped on the right, alongside the total number of prophages found in the given bacterial species.
