# Supplementary figures and images for: Transposable Prophages in Leptospira: An Ancient, Now Diverse, Group Predominant in Causative Agents of Weil’s Disease
Source: Int J Mol Sci. 2021 Dec 14;22(24):13434. doi: 10.3390/ijms222413434 (PMC8705779; doi:10.3390/ijms222413434)

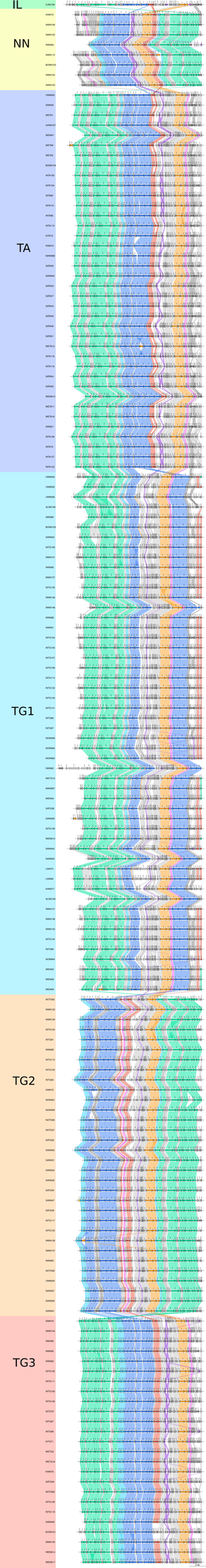

Supplement: Supplementary file 1 [file ijms-22-13434-s001.zip › Figure S4. Genomic maps of the 156 deduplicated prophages.pdf]
